# Carfilzomib-induced thrombotic microangiopathy (TMA) refractory to eculizumab: A case report and literature review

**DOI:** 10.1007/s00277-024-05965-9

**Published:** 2024-08-27

**Authors:** Mina Meseha, Dan Qu, Jill Lykon, David Coffey

**Affiliations:** Myeloma Institute, Sylvester Comprehensive Cancer Center, University of Miami, 1120 NW 14Th Street, Clinical Research Building, Miami, FL USA

**Keywords:** Multiple Myeloma, Carfilzomib, Thrombotic microangiopathy, Eculizumab

## Abstract

This case report describes the clinical course of a patient with relapsed IgA kappa multiple myeloma with high-risk cytogenetics. Initially treated with daratumumab–bortezomib–lenalidomide–dexamethasone (Dara-VRD) then transitioned to lenalidomide maintenance. However, he experienced a relapse and was treated with carfilzomib-based therapy (CFZ) but developed drug-induced thrombotic microangiopathy (DI-TMA). Despite receiving eculizumab and supportive care, the patient's condition worsened, leading to encephalopathy and refractory gastrointestinal bleeding in the setting of persistent thrombocytopenia. Ultimately, the decision was made to transition to comfort-focused care. DI-TMA has been documented with various proteasome inhibitors such as ixazomib and bortezomib. Additionally, other medications such as cyclosporine, tacrolimus, clopidogrel, ticlopidine, and interferon have been associated with DI-TMA as well (Pisoni et al. (Drug Saf 24:491–501, 2001) [18]). Here we discuss a case of carfilzomib-induced TMA (CFZ-TMA) refractory to eculizumab as well as a review of the published literature.

## Introduction

Multiple myeloma (MM) is a plasma cell neoplasm characterized by clonal proliferation of malignant plasma cells in the bone marrow with an abnormal increase of monoclonal protein leading to end-organ damage. Treatment approaches have evolved with the introduction of novel agents and immunotherapies, such as bispecific antibodies and CAR-T cell therapy. However, proteasome inhibitors (PI) are still the mainstay of treatment and come with the risk of adverse events, including TMA, particularly with CFZ, an irreversible PI.

TMA manifests across various clinical conditions including thrombotic thrombocytopenic purpura (TTP), hemolytic uremic syndrome (HUS), atypical HUS (aHUS), DI-TMA, disseminated intravascular coagulopathy (DIC), malignancy, malignant hypertension, and transplant. Several factors contribute to the pathogenesis of DI-TMA, including (a) multiple myeloma as an independent risk factor, (b) germline mutations in the complement alternative pathway (c) reduced VEGF production by renal epithelial cells [1, 2], and (d) immune-mediated or dose-dependent drug toxicity [3]. These mechanisms lead to the common clinical manifestations of TMA syndromes, which include endothelial injury resulting in the formation of microvascular thrombi, microangiopathic hemolysis, and thrombocytopenia [3–5]. Additionally, viral infections have been reported as triggers of acute disease, especially in late-onset CFZ-TMA [6]. This case highlights the challenges in managing CFZ-TMA and the need for other therapeutic options.

## Case report

A 77-year-old male underwent an anemia workup and received a diagnosis of IgA kappa multiple myeloma, Revised International Staging System (R-ISS) stage III. His labs showed serum IgA 4,792 mg/dL with suppressed IgG and IgM, free kappa light chain (FKLC) 16.58 mg/dL, free lambda light chain (FLLC) 0.47 mg/dL, serum creatinine 1.46 mg/dL, serum calcium 9.9 mg/dL, hemoglobin 7.9 g/dL, albumin 3.3 g/dl, B2-microglobulin 12.86 mg/L. Bone marrow biopsy showed 80% plasma cells of overall marrow cellularity. Cytogenetic analysis revealed 1q gain, t(14;16), 13q deletion, trisomy/tetrasomy of chromosomes 1, 4, 11, 13, 14, 15, 16, and 17. Whole body MRI showed no suspicious osseous lesions. He started on daratumumab–bortezomib–lenalidomide–dexamethasone (Dara-VRD) and after eight cycles achieved a complete response (CR) with negative minimal residual disease (MRD) by flow cytometry (10^–5^ sensitivity). Following this, the patient transitioned to lenalidomide maintenance, and autologous stem cell transplant (ASCT) was deferred.

Seven months later, he had a biochemical relapse evidenced by FKLC 15.98 mg/dl and kappa/lambda ratio 10.8. A subsequent bone marrow biopsy demonstrated 70% plasma cells of overall marrow cellularity. The patient’s treatment regimen was adjusted to carfilzomib–pomalidomide–dexamethasone (KPD), consisting of an initial dose of CFZ at 20 mg/m^2^ followed by subsequent doses of 70 mg/m^2^ on days 1, 8, and 15 of each cycle [7]. Pomalidomide was administered at a daily dose of 4 mg for 21 days followed by a 7-day rest period, and dexamethasone 20 mg twice a week. Additionally, he was prescribed apixaban 5 mg twice daily due to an elevated risk of DVT from a history of atrial fibrillation, and acyclovir 400 mg twice daily for viral prophylaxis.

After he had completed the 3rd cycle of KPD, he presented to our institution with 6 days of generalized weakness fatigue, shortness of breath as well as poor appetite, and decreased urinary output. On admission, he had tachypnea (respiratory rate 27–32 breaths/minute), elevated blood pressure (151/77 mmHg), normal heart rate and temperature.

His admission labs showed hemoglobin level decreased from 9.5 g/dL to 7.5 g/dL, platelet count dropped from 161,000/µL to 30,000/µL, creatinine level increased from 1.19 mg/dL to 8.19 mg/dL, with a corresponding decline in estimated glomerular filtration rate (eGFR) from 60 to 6 mL/min/1.73m^2^, and potassium level was 7.3 mmol/l. He was subsequently admitted to the medical ICU and started on intermittent hemodialysis due to persistent hyperkalemia and oliguric acute kidney injury (AKI). A transthoracic echo (TTE) revealed normal cardiac function.

**Fig. 1:**
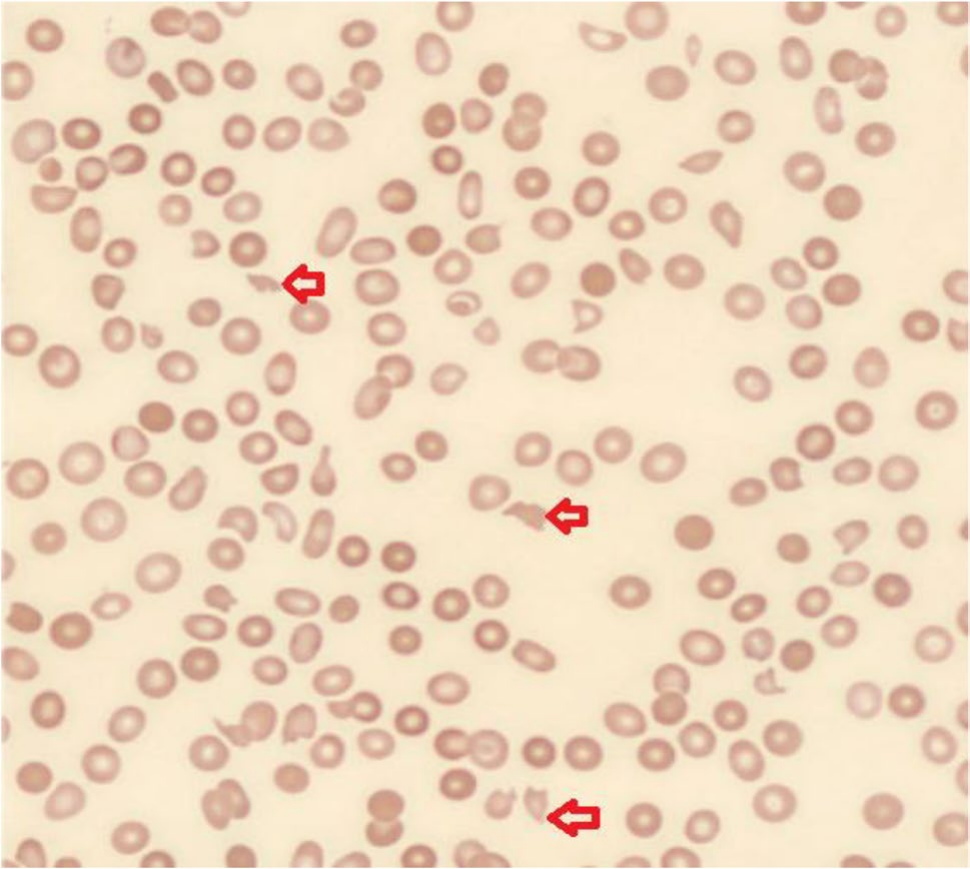
Peripheral smear showing schistocytes (red arrows) (Wright Giemsa stain, 100X oil immersion)

Laboratory findings further indicated evidence of microangiopathic hemolytic anemia (MAHA), with elevated lactate dehydrogenase (LDH) levels at 989 U/L, total bilirubin at 1.7 mg/dL, and haptoglobin levels below 10 mg/dL. Examination of the peripheral blood smear (Fig. 1) revealed the presence of schistocytes, immature myeloid cells, as well as abundant plasmacytoid and lymphocytic elements. Considering the patient's renal failure, thrombocytopenia, and hemolytic anemia, it was suspected that he was experiencing CFZ-TMA. Alternative causes of TMA were investigated and ruled out. The normal coagulation studies and ADAMTS13 activity of 56% (greater than 10%) effectively rule out DIC and TTP. Furthermore, the absence of a history of diarrhea made it unlikely that the TMA was associated with Shiga toxin-producing Escherichia coli (STEC)-related HUS. Complement analysis (C3, C4, and CH50) were within normal ranges further ruling out aHUS. Notably, he was in relapse as indicated by a monoclonal protein of 1.20 g/dL, IgA 4,792 mg/dL, FKLC 64.33 mg/dl, and kappa/lambda ratio 201.03.

**Fig. 2:**
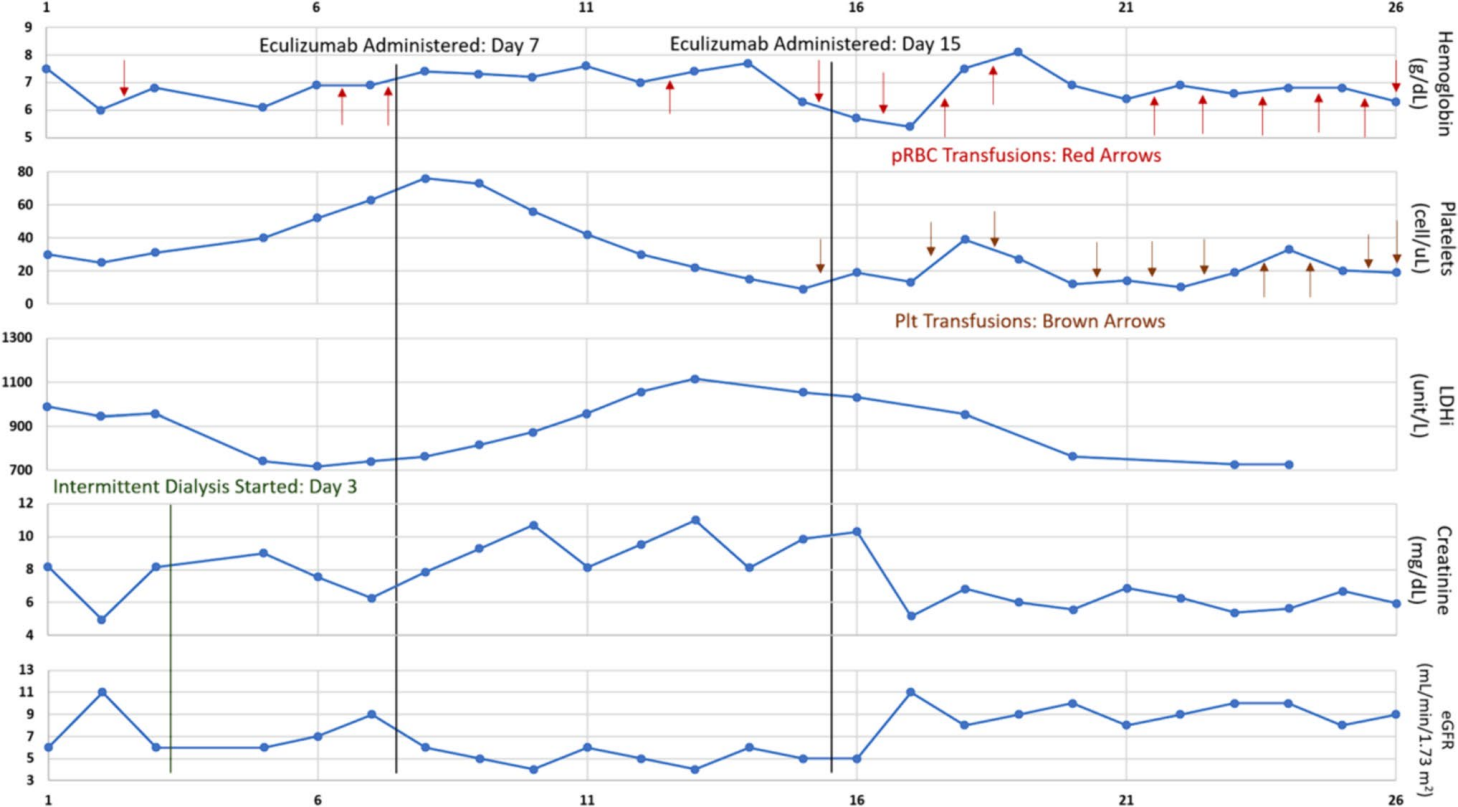
Lab values pre and post-Eculizumab administration

On hospital day 7, a single dose of 900 mg of eculizumab, an anti-complement C5 monoclonal antibody was administered concurrently with the haemophilus B polysaccharide, pneumococcal 23-valent (Pneumovax 23), and meningococcal vaccines. This approach was taken because the risks associated with delaying eculizumab therapy were deemed greater than the risk of developing a serious infection. Additionally, the patient was initiated on prophylactic antibiotic, penicillin 250 mg twice daily, for 2 weeks. Despite administering another dose of eculizumab on day 14, there was no improvement observed in the patient's hemoglobin level, platelet count, or serum creatinine/eGFR (Fig. 2). He continued to rely on supportive measures, including red blood cell and platelet transfusions, as well as hemodialysis. Furthermore, the upper extremities doppler ultrasound revealed acute obstructive DVT in the left axillary and brachial veins. Despite efforts, the patient developed encephalopathy and refractory gastrointestinal bleeding due to ongoing thrombocytopenia. Given these complications, the patient's family decided to transition to comfort-focused care.

## Discussion

CFZ-TMA is an uncommon adverse reaction not initially reported in the original clinical trial. However, A retrospective study documented only 16 cases between 2012 and 2019 [8]. Among 281 newly diagnosed multiple myeloma patients (NDMM) treated with carfilzomib-cyclophosphamide, and dexamethasone in the CARDAMON trial, eight (2.8%) experienced a TMA [9, 10]. Studies have shown that TMA can occur at any stage throughout the treatment course [3, 4, 6, 11]. Despite the wide range of doses at which CFZ is administered, from 20 mg/m^2^ to 70 mg/m^2^, with 56 mg/m^2^ typically considered the therapeutic dose, no specific dose cutoff associated with TMA has been identified [8, 12]. Nevertheless, in a study by Moscvin et al., who observed within their cohort, 7 out of 10 patients with TMA received CFZ at a dose of 56 mg/m^2^ twice weekly indicating dose-dependent drug toxicity [1].

This condition is characterized by MAHA, thrombocytopenia, and acute renal failure. While the precise mechanism remains incompletely understood, it is believed to stem from several factors. Firstly, the effects of multiple myeloma. Secondly, CFZ acts as a ubiquitin–proteasome pathway inhibitor, thereby inhibiting the transcription factor NFκB. This inhibition can disrupt vascular endothelial growth factor (VEGF) production, contributing to complement overproduction. Consequently, this cascade of events can result in endothelial and renal microvasculature damage, microthrombi formation, hemolysis, and platelet consumption. Lastly, CFZ-mediated genetic variations in complement genes such as deletions of complement factor H-related proteins (CFHR) region [1, 2, 8, 13]. Our patient developed TMA following three cycles of 70 mg/m^2^ CFZ once weekly [7]. It is suspected that drug toxicity from this high dose, combined with disease progression, contributed to the development of CFZ-induced TMA.

TMA is typically diagnosed through a process of exclusion of other potential differentials including TTP, HUS, and aHUS. The workup should include a comprehensive assessment. This includes a complete blood count (CBC) to evaluate for anemia and thrombocytopenia. Additionally, a complete metabolic panel (CMP) is essential for assessing AKI. Examination of a peripheral smear is crucial to identify schistocytes, which, when accompanied by elevated LDH levels and low haptoglobin, suggest the presence of MAHA. Nonetheless, renal biopsy to visualize thrombotic angiopathy remains the gold standard for diagnosing TMA. However, it is associated with a significant risk of bleeding, particularly in individuals with thrombocytopenia [2]. Meanwhile, obtaining ADAMTS13 levels is essential to exclude TTP, while complement testing and, if available complement gene analysis are used to rule out aHUS.

The management approach for CFZ-TMA typically includes discontinuing CFZ to prevent further kidney damage. Additionally, early administering of a weekly 900 mg dose of eculizumab, a terminal complement inhibitor, has shown benefit in many case reports [5, 14]. Although the duration may vary depending on the individual case, 4 weeks is recommended. Nevertheless, in a recent large case series study of CFZ-TMA, the patients presented with severe AKI and were treated with eculizumab, yet they showed no apparent improvement in pathophysiology or prognosis [15]. The lack of response to eculizumab could also imply that the pathogenesis may not solely involve the complement pathway.

Some cases have also reported the use of supportive therapies such as plasmapheresis, high-dose glucocorticoid, and hemodialysis [9, 11, 14]. The therapeutic plasma exchange (TPE) is a reasonable intervention in cases of suspected TTP; however, it can be halted if ADAMTS13 activity is found to be normal, thus ruling out TTP [16]. In a study by Fotiou et al., involving 114 CFZ-treated patients, all six patients who developed TMA received plasmapheresis and steroids; rituximab was additionally administered in one patient; but none were treated with eculizumab. Renal function and platelets recovered fully in five patients, whereas one died of sepsis. Notably, none of the patients had progressive myeloma at the time of the event and ADAMTS-13 was evaluated in two patients and was within normal limits [17].

Finally, our case emphasizes the significance of patients’ close monitoring for early signs of TMA during CFZ therapy. It is crucial to consider stopping the medication or early dose adjustments to the treatment as necessary to manage this complication effectively.

## Conclusion

Despite advances in multiple myeloma treatment, managing treatment-related complications remains challenging, particularly in high-risk patients with refractory disease. Therapy-induced TMA, as seen in this case, presents a diagnostic challenge and can lead to serious, potentially life-threatening complications. While eculizumab is a standard treatment for TMA, it was ineffective in halting its progression in this instance. This case highlights the importance of a thorough diagnostic approach for CFZ-TMA and underscores the need for increased vigilance. Healthcare providers should closely monitor patients for early signs of TMA during CFZ therapy, and consider early modifications and dose adjustments to the treatment as necessary.

## Funding declaration

The authors did not receive any specific grant from public, commercial, or not-for-profit funding agencies.

## Data Availability

No datasets were generated or analysed during the current study.

## References

[1] Moscvin M, Liacos CI, Chen T, et al (2023) Mutations in the alternative complement pathway in multiple myeloma patients with carfilzomib-induced thrombotic microangiopathy. Blood Cancer Journal 2023 13:1 13:1–7 10.1038/s41408-023-00802-0 PMC9971259 36849497

[2] Eigbire-Molen O, Hermelin D, Blackall D (2022) Carfilzomib-Induced Thrombotic Microangiopathy: Focus on Pathogenesis. J Med Cases 13:274 35837078 10.14740/jmc3932 PMC9239517

[3] Yui JC, Van Keer J, Weiss BM et al (2016) Proteasome inhibitor associated thrombotic microangiopathy. Am J Hematol 91:E348–E352 27286661 10.1002/ajh.24447

[4] Ponraj R, Bryant A, Dunlop L, Range H, Cobrador C, Ling S, Hsu D (2023) Carfilzomib-induced thrombotic microangiopathy (TMA): an under-recognised spectrum of disease from microangiopathic haemolysis to subclinical TMA. Blood Cancer Journal 2023 13:1 13:1–4 10.1038/s41408-023-00885-9 PMC10371986 37495597

[5] Zafar A, Lim MY, Abou-Ismail MY (2023) Eculizumab in the management of drug-induced thrombotic microangiopathy: A scoping review of the literature. Thromb Res 224:73–79 36871347 10.1016/j.thromres.2023.02.012

[6] Pallotti F, Queffeulou C, Bellal M, Jean-Jacques B, Gac AC, Chatelet V, Boyer A, Gueutin V (2022) Carfilzomib-Induced Thrombotic Microangiopathy Treated with Eculizumab: A Case Report and Rapid Literature Review. Kidney and Dialysis 2022, Vol 2, Pages 625–637 2:625–637

[7] Moreau P, Mateos MV, Berenson JR, Weisel K, Lazzaro A, Song K, Dimopoulos MA, Huang M, Zahlten-Kumeli A, Stewart AK (2018) Once weekly versus twice weekly carfilzomib dosing in patients with relapsed and refractory multiple myeloma (A.R.R.O.W.): interim analysis results of a randomised, phase 3 study. Lancet Oncol 19:953–964 29866475 10.1016/S1470-2045(18)30354-1

[8] Jindal N, Jandial A, Jain A, Lad D, Prakash G, Khadwal A, Nada R, Sethi J, Ahluwalia J, Malhotra P (2020) Carfilzomib-induced thrombotic microangiopathy: A case based review. Hematol Oncol Stem Cell Ther 16:426–431 10.1016/j.hemonc.2020.07.001 32735793

[9] Terao T, Tsushima T, Miura D, Ikeda D, Fukumoto A, Kuzume A, Tabata R, Narita K, Takeuchi M, Matsue K (2022) Carfilzomib-induced thrombotic microangiopathy is underestimated in clinical practice: A report of five patients and literature review. Leuk Lymphoma 63:1102–1110 35373680 10.1080/10428194.2022.2057485

[10] Camilleri M, Cuadrado M, Phillips E et al (2021) Thrombotic microangiopathy in untreated myeloma patients receiving carfilzomib, cyclophosphamide and dexamethasone on the CARDAMON study. Br J Haematol 193:750–760 33650100 10.1111/bjh.17377 PMC11497300

[11] Haddadin M, Al-Sadawi M, Madanat S, Tam E, Taiwo E, Luhrs C, Mcfarlane SI (2019) Late Presentation of Carfilzomib Associated Thrombotic Microangiopathy. American Journal of Medical Case Reports, Vol 7, 2019, Pages 240–243 7:240–243

[12] Kastritis E, Laina A, Georgiopoulos G, et al (2021) Carfilzomib-induced endothelial dysfunction, recovery of proteasome activity, and prediction of cardiovascular complications: a prospective study. Leukemia 2021 35:5 35:1418–1427 10.1038/s41375-021-01141-4 33589757

[13] Portuguese AJ, Lipe B (2018) Carfilzomib-induced aHUS responds to early eculizumab and may be associated with heterozygous CFHR3-CFHR1 deletion. Blood Adv 2:3443–3446 30518536 10.1182/bloodadvances.2018027532 PMC6290108

[14] Rassner M, Baur R, Wäsch R, Schiffer M, Schneider J, Mackensen A, Engelhardt M (2021) Two cases of carfilzomib-induced thrombotic microangiopathy successfully treated with Eculizumab in multiple myeloma. BMC Nephrol 22:1–7 33461512 10.1186/s12882-020-02226-5 PMC7814610

[15] Joseph A, Harel S, Mesnard L et al (2024) Carfilzomib-associated thrombotic microangiopathy: clinical features and outcomes. Nephrol Dial Transplant. 10.1093/NDT/GFAE096 38658194 10.1093/ndt/gfae096

[16] Qaqish I, Schlam IM, Chakkera HA, Fonseca R, Adamski J (2016) Carfilzomib: A cause of drug associated thrombotic microangiopathy. Transfus Apheres Sci 54:401–404 10.1016/j.transci.2016.03.002 27017313

[17] Fotiou D, Roussou M, Gakiopoulou C, et al (2020) Carfilzomib-associated renal toxicity is common and unpredictable: a comprehensive analysis of 114 multiple myeloma patients. Blood Cancer Journal 2020 10:11 10:1–5 10.1038/s41408-020-00381-4 PMC7642386 33149167

[18] Pisoni R, Ruggenenti P, Remuzzi G (2001) Drug-induced thrombotic microangiopathy: Incidence, prevention and management. Drug Saf 24:491–501 11444722 10.2165/00002018-200124070-00002

